# Supplementary figures and images for: The Longitudinal Analysis on the Anti-SARS-CoV-2 Antibodies among Healthcare Workers in Poland—Before and after BNT126b2 mRNA COVID-19 Vaccination
Source: Vaccines (Basel). 2022 Sep 20;10(10):1576. doi: 10.3390/vaccines10101576 (PMC9607217; doi:10.3390/vaccines10101576)

## Slide 1
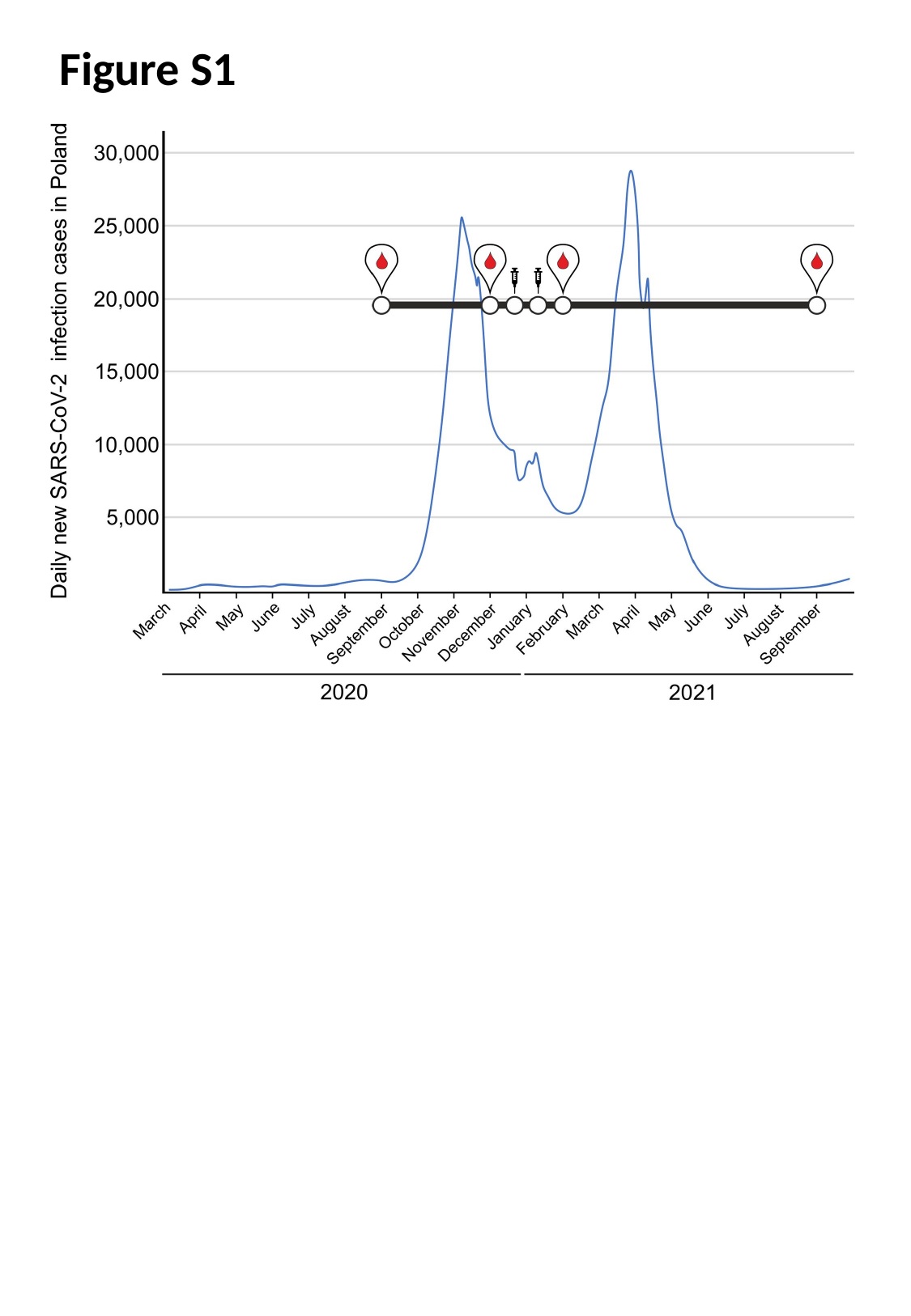

Figure S1

## Slide 2
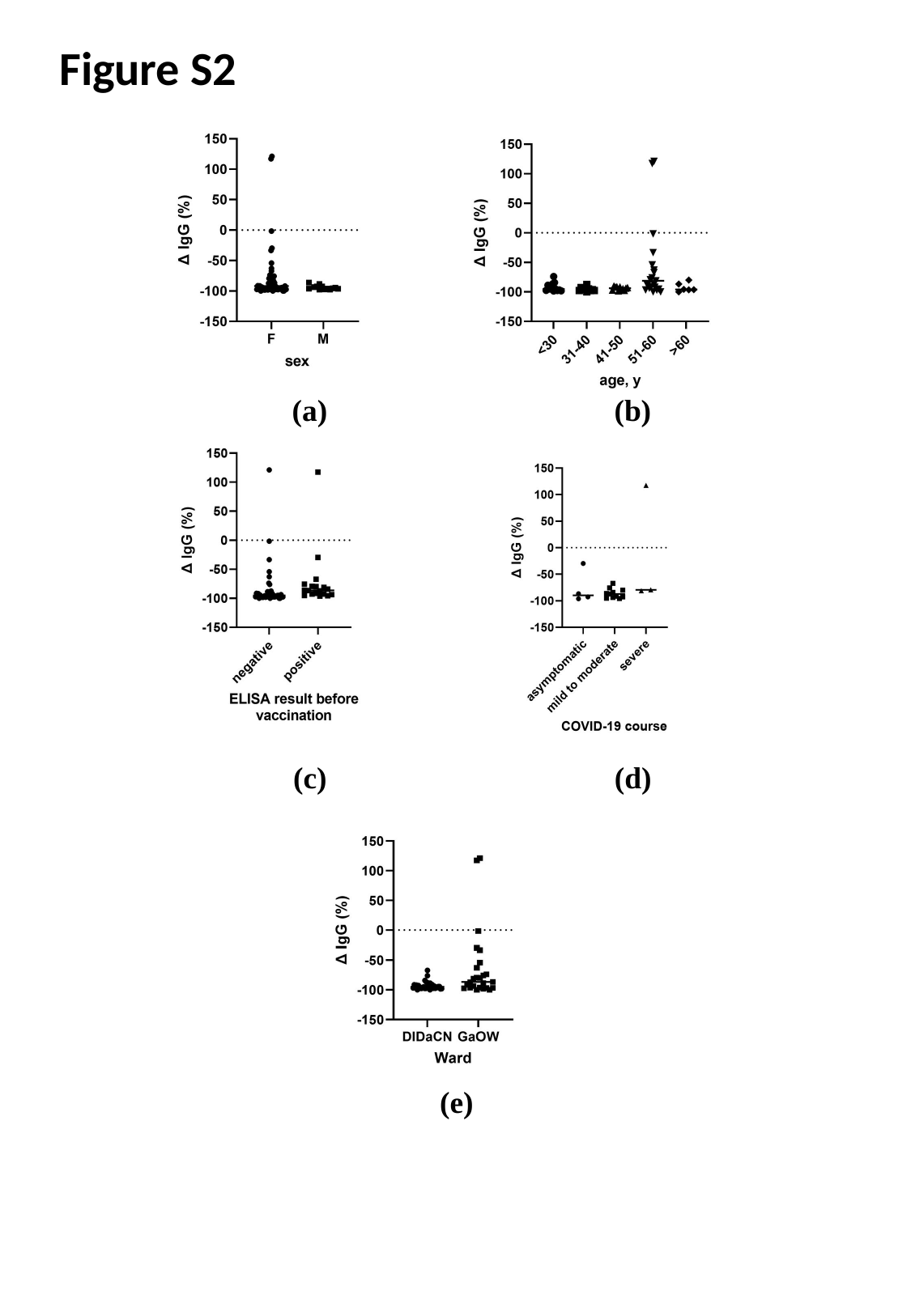

Figure S2
(a)
(b)
(c)
(d)
(e)

Supplement: Supplementary file 1 [file vaccines-10-01576-s001.zip › vaccines-1863308-supplementary.pptx]
